# A Novel Inherently Flame-Retardant Composite Based on Zinc Alginate/Nano-Cu_2_O

**DOI:** 10.3390/polym11101575

**Published:** 2019-09-27

**Authors:** Peng Xu, Peiyuan Shao, Qing Zhang, Wen Cheng, Zichao Li, Qun Li

**Affiliations:** 1 College of Chemical Science and Engineering, Qingdao University, Qingdao 266071, China; X1627271005@163.com (P.X.); 2017020828@qdu.edu.cn (P.S.); 20180205203@qdu.edu.cn (Q.Z.); wencheney2014@163.com (W.C.); 2 Institute of Advanced Cross-Field Science, College of Life Sciences, Qingdao University, Qingdao 266071, China

**Keywords:** zinc alginate, nano-Cu_2_O, flame retardancy, pyrolysis, thermal degradation

## Abstract

A novel flame-retardant composite material based on zinc alginate (ZnAlg) and nano-cuprous oxide (Cu_2_O) was prepared through a simple, eco-friendly freeze-drying process and a sol-gel method. The composites were characterized and their combustion and flammability behavior were tested. The composites had high thermal stability and achieved nearly non-flammability with a limiting oxygen index (LOI) of 58. The results show remarkable improvement of flame-retardant properties in the ZnAlg/Cu_2_O composites, compared to ZnAlg. Furthermore, the pyrolysis behavior was determined by pyrolysis–gas chromatography–mass spectrometry (Py-GC-MS) and the flame-retardant mechanism was proposed based on the combined experimental results. The prepared composites show promising application prospects in building materials and the textile industry.

## 1. Introduction

Sodium alginate (NaAlg) is a hydrophilic and biodegradable linear polysaccharide copolymer consisting of two different ratios of spatially different 1,4-linked α-L guluronic acid and β-D mannuronic acid repeating unit composition [1,2,3]. The unique properties of sodium alginate are biological origin, non-toxic, hydrophilic, biocompatible, biodegradable and low-cost, making it highly applicable in various fields [4,5]. The most important characteristic of NaAlg is that it can react with polyvalent metal cations to form strong gels or insoluble polymers [6,7,8], thereby improving water resistance, mechanical properties, barrier properties, cohesiveness and rigidity during cross-linking with multivalent cations [9]. Zinc alginate is a material with excellent biological activity and is widely used in flame-retardant and antibacterial applications [10,11].

Nanomaterials have a wide range of applications in flame retardancy [12]. Norouzi et al. investigated the effect of the addition of different nanomaterials on the flame retardancy of textiles and found that most nanoparticles can improve the thermal stability and flame-resistant textile polymers [13]. Nanoparticles of different kinds of materials, such as silver, titanium dioxide and zinc oxide, have been reported to be functionalized for fibers and fabrics to achieve significantly improved products with new macroscopic properties [14]. Nano-Cu_2_O is a new type of p-type oxide semiconductor material that can be excited by visible light. It has an active electron-hole pair system and exhibits good catalytic activities [15,16,17]. In addition, it shows excellent adsorption properties and low-temperature paramagnetism, and has potential applications in organic synthesis, photoelectric conversion, new energy, photolysis of water, dye bleaching, sterilization, superconductivity and other fields [17,18,19,20,21,22,23]. However, few studies have been focused on flame retardancy of materials based on nano-Cu_2_O to date.

In this study, ZnAlg/Cu_2_O composite materials were prepared by a simple, economical and environmentally friendly sol-gel method. The composites were characterized by scanning electron microscopy (SEM), X-ray diffractometry (XRD), X-ray photoelectron spectroscopy (XPS), Fourier transform infrared spectrum (FTIR), and thermogravimetric analysis (TG). The combustion and flammability behaviors of the composites were assessed by the limiting oxygen index (LOI), vertical burning rate (UL-94) and cone calorimetry (CONE). Additionally, the pyrolysis products of the composites, SEM of the char residue, were investigated to reveal the flame-retardant mechanism of the prepared materials.

## 2. Materials and Methods

### 2.1. Materials

NaAlg was supplied by the Institute of Photosynthetic Fine Chemicals (Tianjin, China). Cupric sulfate (CuSO_4_·5H_2_O) was purchased from Tianjin Beichen Founder Reagent Factory (Tianjin, China). Zinc acetate dehydrate (C_4_H_6_O_4_Zn·2H_2_O) and L-Ascorbic acid (VC) were obtained from Sinopharm Chemical Reagent Co., Ltd. (Shanghai, China). Ammonia solution (NH_3_·H_2_O) was purchased from Kant Chemical Co., Ltd. (Laiyang, China). All the chemicals were not further purified and all the solutions were prepared using deionized water.

### 2.2. Preparation of ZnAlg

Firstly, 20.00 g of NaAlg was added in 500 mL deionized water and stirred to form a uniform sol. After standing for 24 h, the NaAlg sol was shaped in a square mold. Then, 3 wt % C_4_H_6_O_4_Zn·2H_2_O solution was added and crosslinked with NaAlg for 48 h. The final product was washed several times with deionized water and dried in a freeze dryer (FD-1A-50, Billing Instrument Manufacturing Co., Ltd, Shanghai, China).

### 2.3. Preparation of ZnAlg/Cu_2_O Composites

**Scheme 1:**
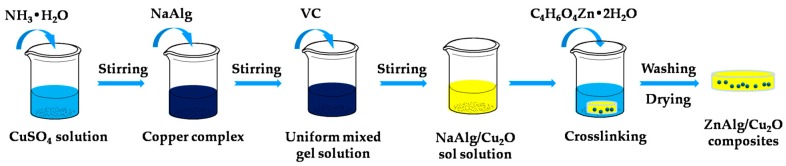
Preparation of ZnAlg/Cu_2_O composite materials.

The ZnAlg/Cu_2_O composites were prepared as illustrated in Scheme 1. Briefly, CuSO_4_·5H_2_O (2.34 g) was dissolved in 450 mL deionized water and stirred to uniform dispersion in deionized water. Then, 7.5 mL of NH_3_·H_2_O was added and stirred to form a copper complex solution. Subsequently, 20.00 g of NaAlg was slowly added and stirred to form a uniform mixed gel solution. After standing for 24 h, 50 mL 13.67 wt % VC solution was slowly added to the mixed gel solution and stirred until it turned yellow to produce the NaAlg/Cu_2_O mixed gel solution. The as-prepared gel solution was poured and shaped in a square mold, followed by the addition of 3 wt % C_4_H_6_O_4_Zn·2H_2_O (500 mL) solution. Crosslinked reaction was proceeded for 24 h to obtain the ZnAlg/Cu_2_O composites. The final product was washed several times with deionized water and dried in a freeze dryer for further use.

### 2.4. Measurements

The morphology and microstructure of the samples were examined by SEM (SIGMA, Zeiss, Oberkochen, Germany). All samples on the surface of the study were sprayed with gold.

The XRD of the prepared sample was carried out on a D8 Advance diffractometer (Bruker, Karlsruhe, Germany). The sample was scanned in 2θ to 90° in continuous mode.

The XPS was recorded on an ESCALAB 250Xi (Thermo Fisher Scientific, Waltham, MA, USA). The orbit of Cu element is 2p orbital.

The chemical bonds in the samples were determined by FTIR spectroscopy (NICOLET iS50, Thermo Fisher Scientific, Madison, WI, USA). The wavenumbers ranged from 4000 to 500 cm^−1^.

The LOI tests were performed on a digital limiting oxygen index tester (LFY-606B, Shandong Textile Science Research Institute, Jinan, China) according to the standard method ISO 4589-1:1996. The size of all samples was 130 mm × 10 mm.

The UL-94 tests were performed on a LFY-601A vertical burning rate tester (Shandong Textile Science Research Institute, Jinan, China) according to the standard method ANST/UL-94-1985. The size of all samples was 130 mm × 13 mm × 5 mm.

TG was carried out on an SDT Q600 thermogravimetric analyzer (TA Instrument, New Castle, DE, USA), and the samples were raised from 25 to 900 °C at a heating rate of 10 °C /min in ambient environment as well as nitrogen atmosphere.

The combustion properties of the samples were measured using an FTT-0242 cone calorimeter device (Fire Testing Technology, East Grinstead, UK) under an external heat flux of 35 kW/m^2^ according to standard ISO 5660. The dimensions of all samples were 100 mm × 100 mm × 3 mm.

Py-GC-MS was performed by a thermal cracker (EGA/PY-3030D, Frontier, Koriyama, Japan) and gas chromatography–mass spectrometer (TRACE 1310-ISQLT, Thermo Fisher Scientific, Waltham, MA, USA). The pyrolysis temperature was 250, 450, and 750 °C, and the carrier gas was He. The GC temperature program was started at 50 °C, and stayed in the pose for 3 min, and then rose to 300 °C at the speed of 20 °C/min.

## 3. Results and Discussion

### 3.1. Characterizations

**Figure 1:**
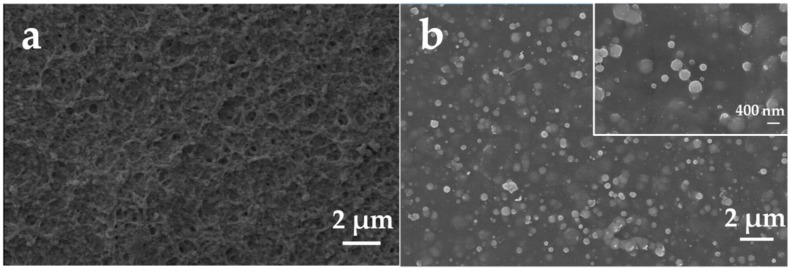
SEM images of (**a**) ZnAlg and (**b**) ZnAlg/Cu_2_O composites. The inset in (**b**) is its magnified image.

The morphology of the prepared samples was investigated by SEM and the images are shown in Figure 1. It can be observed from Figure 1a that the surface of ZnAlg showed a network structure and was relatively rough. As can be seen from Figure 1b, spherical nano-Cu_2_O was embedded in ZnAlg, ranging from 100–400 nm in size, indicating a successful preparation of the ZnAlg/Cu_2_O composites. Moreover, the addition of nano-Cu_2_O made the surface of ZnAlg smoother.

**Figure 2:**
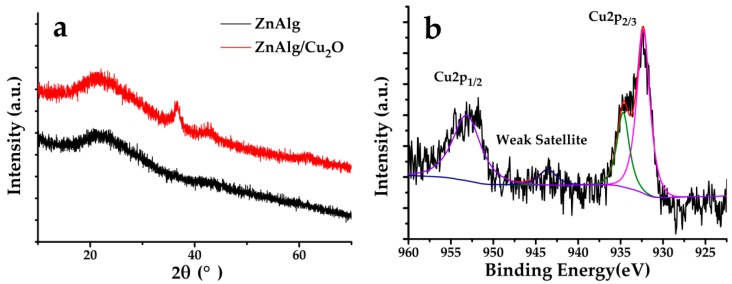
(**a**) X-ray diffractometry (XRD) patterns of ZnAlg and ZnAlg/Cu_2_O and (**b**) main and satellite peaks of Cu 2p3/2 and Cu 2p1/2 of the X-ray photoelectron spectroscopy (XPS) spectra for ZnAlg/Cu_2_O.

The diffraction peaks at 36.46°, 42.34° and 61.43° of the ZnAlg/Cu_2_O samples can be seen in Figure 2a, which can be assigned to Cu_2_O, indicating a successful introduction of Cu_2_O for the sample preparation [24]. Moreover, no other obvious peaks can be observed in the images, suggesting high purity of Cu_2_O in the material.

Figure 2b displays the XPS spectrum of the ZnAlg/Cu_2_O composites. As seen, the two peaks at 932.7 and 953.2 eV can be corresponded to the Cu 2p3/2 and Cu 2p1/2 binding energies of Cu_2_O. The results reflect the high purity of Cu_2_O in ZnAlg/Cu_2_O, which is in good agreement with that of previous reports [25,26,27,28]. Moreover, as shown in Figure S1 (in Supplementary Materials), the content of Cu was 0.75 at. %, while that of Zn was 2.17 at. %.

**Figure 3:**
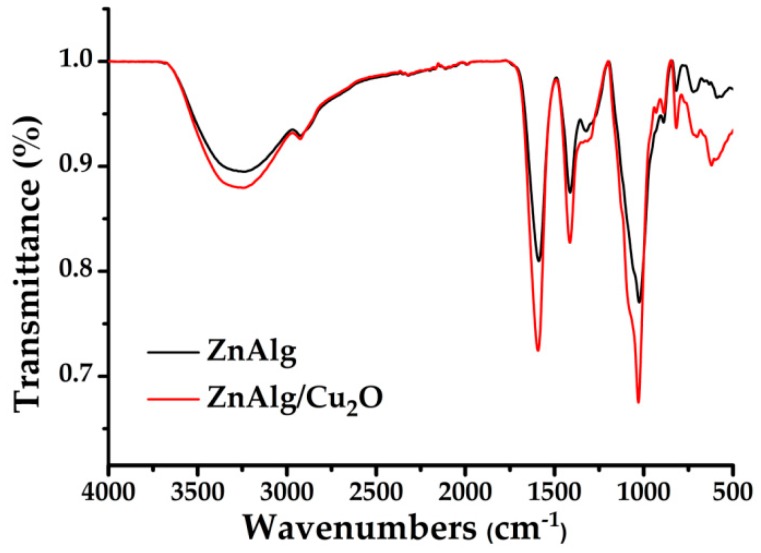
Fourier transform infrared spectrum (FTIR) spectra of ZnAlg and ZnAlg/Cu_2_O.

The FTIR spectra of the samples are shown in Figure 3. A series of characteristic absorption peaks were observed, of which 3240 cm^−1^ (O–H stretching vibration), 2923 cm^−1^ (–CH_2_), 1584 cm^−1^ (C=O stretching vibration) [29], 1429 cm^−1^ (symmetric and asymmetric vibrations of –COO), 1029 and 914 cm^−1^ (symmetric and asymmetric vibrations of C–O–C), respectively. No significant differences between the infrared peaks of ZnAlg/Cu_2_O and ZnAlg can be noticed, indicating that the addition of Cu_2_O did not break the structure of ZnAlg [30,31].

### 3.2. Thermal Stability

**Figure 4:**
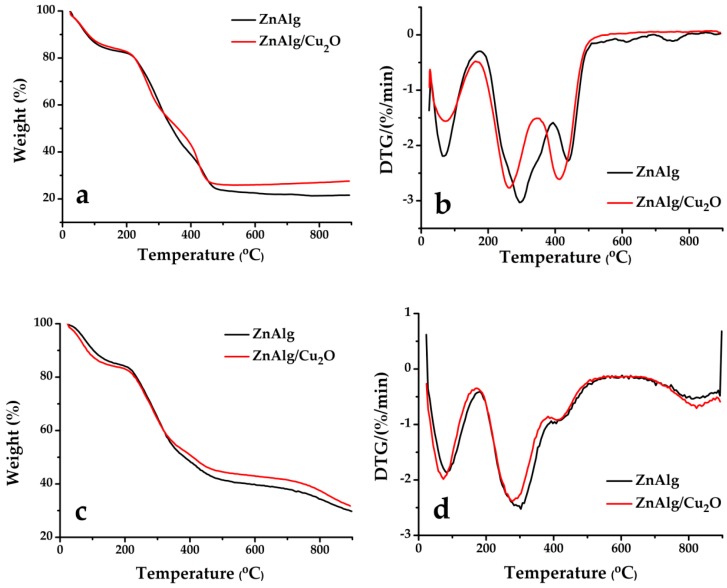
(**a**) Thermogravimetric analysis (TG) and (**b**) derivative thermogravimetry (DTG) curves of ZnAlg/Cu_2_O and ZnAlg under air; (**c**) TG and (**d**) DTG curves of ZnAlg/Cu_2_O and ZnAlg under nitrogen.

The thermal stability of the materials was tested in the air as well as the nitrogen atmosphere, and the results show both materials had similar degradation trends. It can be observed from Figure 4a,b that, under air, from the start of heating to 200 °C, there was about 18% weight loss, which was caused by evaporation of free water and crystal water in the materials, suggesting high natural moisture regain of the materials [31]. From 200 to 450 °C, the masses of ZnAlg/Cu_2_O and ZnAlg were dropped sharply, which was mainly due to the cleavage of glycosidic bonds in the alginate, as well as decarboxylation and decarbonylation [32]. Over 450 °C, the mass of ZnAlg remained nearly unchanged, and the phase change mainly occurred during this period [8], while the mass of ZnAlg/Cu_2_O increased, which was caused by the oxidation of Cu_2_O to CuO (2Cu_2_O + O_2_ = 4CuO) [33,34]. The results show that ZnAlg/Cu_2_O produced more residues than ZnAlg, which was about 6% higher, indicating better thermal stability of ZnAlg/Cu_2_O [35,36].

In nitrogen atmosphere, displayed in Figure 4c,d, from the beginning to 200 °C, the ZnAlg curve ran above the ZnAlg/Cu_2_O curve, indicating that the natural moisture regain of ZnAlg/Cu_2_O was higher, and the addition of Cu_2_O accelerated the dehydration of ZnAlg. At 200–300 °C, ZnAlg and ZnAlg/Cu_2_O almost lost weight simultaneously, suggesting that Cu_2_O did not play its part within this period. After 450 °C, the mass of ZnAlg/Cu_2_O continued to decrease in the nitrogen environment, while the mass of ZnAlg/Cu_2_O increased under air, which further confirmed the oxidation of Cu_2_O. The weight loss rate of ZnAlg/Cu_2_O was significantly lower than that of ZnAlg, suggesting that O_2_ can be absorbed during the oxidation of Cu_2_O to CuO, thereby suppressing combustion [35]. It can be inferred that CuO can act as dust on ZnAlg, to absorb heat and dissipate in the combustion area, thus inhibited the degradation of ZnAlg [31].

### 3.3. Flame Retartancy

The flame-retardant properties of the two prepared samples were investigated by LOI and UL-94 tests and the test data are listed in Table 1. As seen, both ZnAlg/Cu_2_O and ZnAlg had a high LOI. Moreover, the LOI of ZnAlg was increased from 49 to 58, indicating that the introduction of Cu_2_O can efficiently improve the flame retardancy of the basic material. ZnAlg/Cu_2_O was extinguished within 10 s after being ignited in the vertical burning test, and it passed the test without any dripping, which is favorable for its further real application.

### 3.4. Combustion Behavior

**Figure 5:**
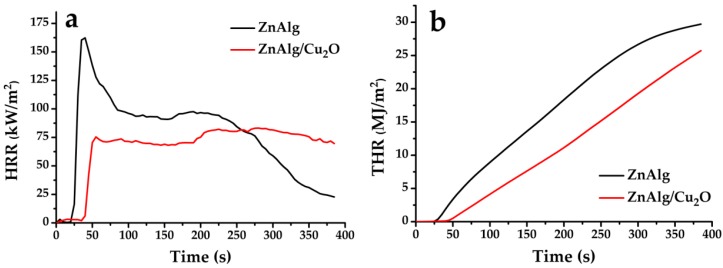

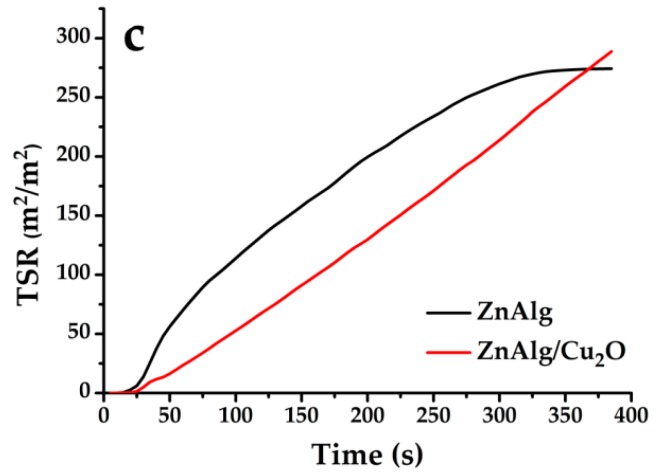
(**a**) HRR, (**b**) THR and (**c**) TSR curves of ZnAlg and ZnAlg/Cu_2_O changing with time.

CONE has been widely employed to evaluate the combustion behavior of the polymers [37,38]. The most important flame-retardant parameters that CONE can give out are ignition time, heat release rate (HRR) and total heat release (THR), as these parameters may be related to flame growth and toxic gas emissions during combustion [1,39]. The detailed data are collected in Table 1 and the related curves are displayed in Figure 5. The ignition time of ZnAlg was 22 s, while that of ZnAlg/Cu_2_O was 37 s, indicating that the polymers were more difficult to be burnt. HRR is an important parameter for evaluating fire safety [40,41]. As shown in Figure 5a, the HRR value of ZnAlg/Cu_2_O was much lower than that of ZnAlg in the most part of the combustion processes. Moreover, the peak heat release rate (PHRR) of ZnAlg/Cu_2_O was only 83.18 kW/m^2^, compared to that of ZnAlg which was 162.31 kW/m^2^. As seen in Table 1, ZnAlg was burnt completely at 387 s, while ZnAlg/Cu_2_O continued and completed at 610 s, indicating that ZnAlg was more easily flammable than ZnAlg/Cu_2_O, and that the catalytic and carbon formation function of Cu_2_O reduced the combustion properties of the material. It can be observed from Figure 5b,c that the THR and TSR values of the two samples were increased with time. ZnAlg/Cu_2_O showed lower THR and TSR values, suggesting lower heat release and less smoke release than ZnAlg. This may be attributed to the fact that O_2_ can be absorbed during the oxidation process of Cu_2_O to CuO at high temperatures. The generated CuO can act as a dust layer on the ZnAlg, absorbing heat in the combustion zone and blocking the release of smoke. In addition, the residual amount of ZnAlg/Cu_2_O was higher than that of ZnAlg. Combining these results, the ZnAlg/Cu_2_O composites showed remarkably better flame retardancy than ZnAlg.

### 3.5. Flame-Retardant Mechanism

**Figure 6:**
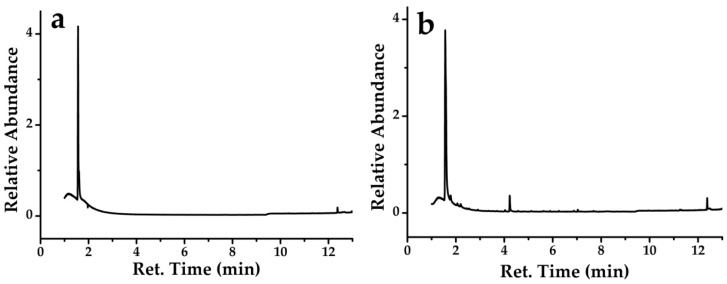

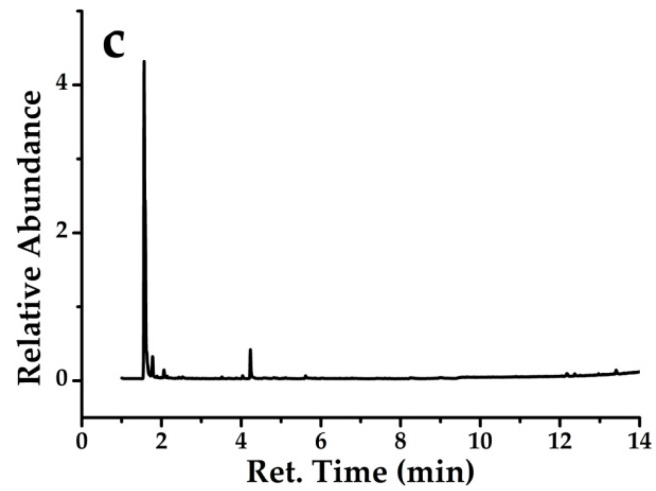
Py-GC-MS spectra of ZnAlg/Cu_2_O at 250 °C (**a**), 450 °C (**b**) and 750 °C (**c**).

Py-GC-MS is mainly used to analyze the main components of gaseous products produced during the pyrolysis of materials [42]. The chromatograms of ZnAlg/Cu_2_O obtained by Py-GC-MS at 250, 450 and 750 °C are shown in Figure 6, respectively, and the major pyrolysis products determined by comparison with the NIST library are listed in Table 2. The main compounds produced at 450 °C were CO_2_, CO, furfural, acetic acid, propanoicacid, 2-oxo-, n-hexadecanoic acid and 9-hexadecenoic acid. As can be seen, the main products can be divided into four groups, including CO, CO_2_, organic acids and aldehydes, respectively [43]. As the temperature increased, more aromatic hydrocarbons, alcohols and ketones were produced. Figure S2 (in Supplementary Materials) displays the chromatograms of ZnAlg obtained by Py-GC-MS at 750 °C, and the main pyrolysis products identified based on the comparison with the NIST library are listed in Table S1 (in Supplementary Materials). Compared with ZnAlg, ZnAlg/Cu_2_O produced less compounds, indicating that the addition of Cu_2_O increased the char yield of ZnAlg. The formed carbon layer covered the surface of the matrix material, hindering the heat transfer and exchange of combustible gases between the matrix material and the outside. It had well protective function to the matrix material, thereby can improve its thermal stability [30,31].

**Scheme 2:**
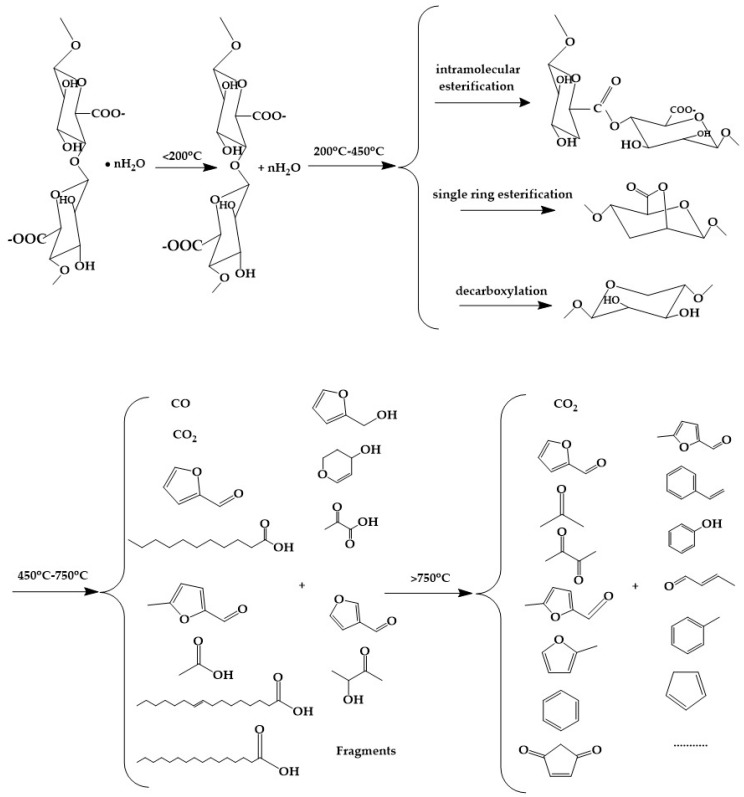
Proposed thermal degradation mechanism of ZnAlg/Cu_2_O.

According to the test results of Py-GC-MS, the proposed thermal degradation mechanisms of ZnAlg/Cu_2_O are depicted in Scheme 2. Below 200 °C, the decomposition products of the composites contained a large amount of H_2_O, indicating massive loss of free and bound water. From 200 to 450 °C, the glycosidic bond of ZnAlg/Cu_2_O was broken, while esterification and decarboxylation reaction occurred. Furthermore, as the degradation mechanism is complicated, thus an intramolecular esterification process (or between different rings) can more likely occur during the process and contribute to the cross-linking of the organic material to form carbon layers [44,45]. From 450 to 750 °C, dehydroxylation, decarboxylation, esterification, decarbonylation, rupture and rearrangement were believed to contribute to the improvement of char formation, releasing CO_2_, CO, organic acids and aldehydes, such as furfural, acetic acid, propanoicacid, 2-oxo-, n-hexadecanoic acid, 9-hexadecenoic acid and etc. The above chemical reactions may be catalyzed by CuO. At 750 °C, the residue may be further degraded by condensation reaction. Cu_2_O accelerated the dehydration of ZnAlg at low temperatures, which caused the formation of a carbon barrier. Furthermore, O_2_ can be absorbed during the Cu_2_O oxidation to CuO at high temperatures. The generated CuO can act as dust covered on ZnAlg, absorbing heat and dissipating in the combustion zone. According to the theory of wall effect, the flame cannot grow if dust is sufficient in the air [46]. Therefore, it can be inferred from the combined results that the introduction of Cu_2_O to ZnAlg significantly improved its flame retardancy.

## 4. Conclusions

A new intrinsic flame-retardant composite based on ZnAlg/nano-Cu_2_O was first prepared through a simple, economical and environmentally friendly method. The prepared material exhibited excellent flame-retardant properties. It was found that the addition of nano-Cu_2_O can promote the conversion of ZnAlg to carbon-based materials. Moreover, O_2_ can be absorbed during the oxidation of Cu_2_O to CuO at high temperatures. The CuO in the polymer can act as dust on the substrate material, covering on its surface when burnt. Thus, it can hinder the heat transfer between the matrix material and the outside and provided good protection on the matrix material. Therefore, the prepared materials can be highly prospective for application in building insulation materials and textile industry.

## Figures and Tables

**Table 1:** Limiting oxygen index (LOI), vertical burning rate (UL-94) testing results and combustion parameters of the prepared samples.

Sample	LOI (%)	UL-94	Time to Ignition (s)	Time to Flameout (s)	THR (MJ/m^2^)	PHRR (kW/m^2^)	TSR (m^2^/m^2^)	Residue (%)
ZnAlg	49	V-0	22	387	29.71	162.31	274.28	51.29
ZnAlg/Cu_2_O	58	V-0	37	610	25.71	83.18	288.75	56.74

PHRR: peak heat release rate; THR: total heat release; TSR: total smoke release.

**Table 2:** Pyrolysis products of ZnAlg/Cu_2_O at different temperatures.

Molecular Structure Name of Compound		T = 250 °C		T = 450 °C		T = 750 °C	
		Time	Area	Time	Area	Time	Area
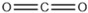	carbon dioxide	1.87	2.03	1.57	71.37	1.57	74.46
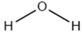	water	1.57	68.52	-	-	-	-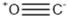	carbon monoxide	1.15	18.36	1.26	6.74	-	-
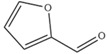	furfural	-	-	4.23	3.88	4.23	5.67
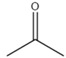	acetone	-	-	-	-	1.78	3.54
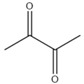	2,3-butanedione	-	-	-	-	2.06	1.24
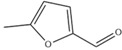	2-furancarboxaldehyde,5-methyl	-	-	5.62	0.27	5.62	0.74
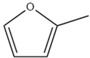	furan,2-methyl-	-	-	-	-	2.14	0.30
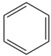	benzene	-	-	-	-	2.53	0.50
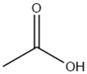	acetic acid	-	-	2.20	1.03	-	-
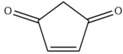	4-cyclopentene-1,3-dione	-	-	-	-	4.83	0.37
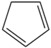	1,3-cyclopentadiene	-	-	-	-	1.89	0.14
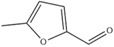	2-furancarboxaldehyde,5-methyl	-	-	-	-	5.12	0.73
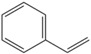	styrene	-	-	-	-	4.90	0.12
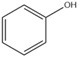	phenol	-	-	-	-	6.04	0.19
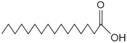	n-hexadecanoic acid	12.38	1.68	12.38	2.25	-	-
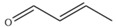	2-butenal, (E)-	-	-	-	-	2.44	0.27
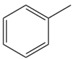	toluene	-	-	-	-	3.51	0.34
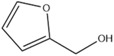	2-furanmethanol	-	-	7.03	0.46	-	-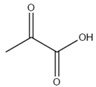	propanoicacid,2-oxo-	-	-	1.79	1.33	-	-
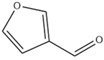	3-furaldehyde	-	-	4.03	0.48	-	-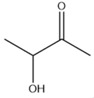	acetoin	-	-	2.91	0.23	-	-
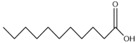	undecanoic acid	-	-	8.26	0.29	-	-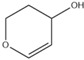	2h-pyran,3,4-dihydro-4-hydroxy	-	-	11.26	0.30	-	-
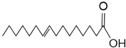	9-hexadecenoicacid	-	-	9.60	0.91	-	-

## Supplementary Materials

The following are available online at https://www.mdpi.com/2073-4360/11/10/1575/s1, Figure S1: The XPS spectra of survey for ZnAlg/Cu_2_O; Figure S2: Py-GC-MS spectra of ZnAlg at 750 °C; Table S1: Pyrolysis products of ZnAlg at 750 °C.
