# Supplementary material for: A Novel Inherently Flame-Retardant Composite Based on Zinc Alginate/Nano-Cu2O
Source: Polymers (Basel). 2019 Sep 27;11(10):1575. doi: 10.3390/polym11101575 (PMC6835664; doi:10.3390/polym11101575)
Supplement: Supplementary file 1 [file polymers-11-01575-s001.pdf]

Supplementary Information

A Novel Inherently Flame-Retardant Composite Based on Zinc Alginate/Nano-Cu_2O

Peng Xu [1], Peiyuan Shao [1], Qing Zhang [1], Wen Cheng [1], Zichao Li [2,*] and Qun Li [1,*]

[1] College of Chemical Science and Engineering, Qingdao University, Qingdao 266071, China
[2] Institute of Advanced Cross-Field Science, College of Life Sciences, Qingdao University, Qingdao 266071, China
* Correspondence: qunli@qdu.edu.cn (Q.L.), zichaoli@qdu.edu.cn (Z.L.); Tel.: +86-532-8595-0705(Q.L.)

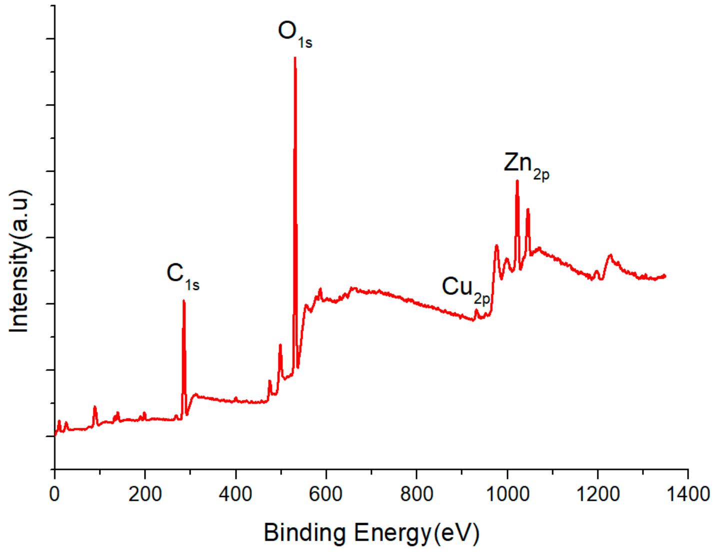

Figure S1. The XPS spectra of survey for ZnAlg/Cu_2O.

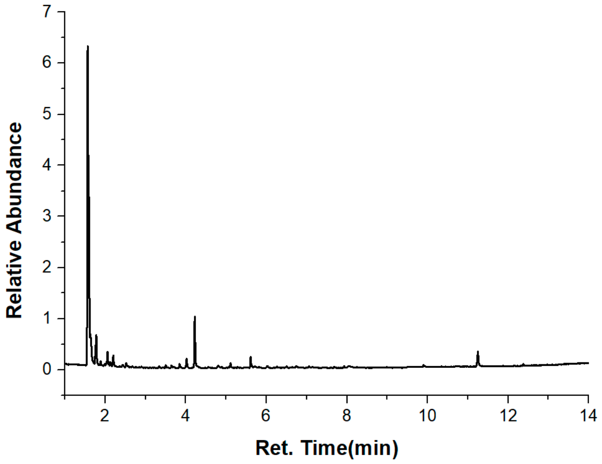

Figure S2. Py-GC-MS spectra of ZnAlg at 750 °C

Table S1. Pyrolysis products of ZnAlg at 750 °C

Molecular structure	Name of compound	T=750 °C	
		**Time**	**Area**
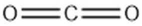	carbon dioxide	1.57	63.56
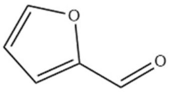	furfural	4.23	7.95
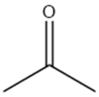	acetone	1.78	4.50
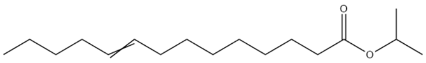	i-Propyl 9-tetradecenoate	11.25	2.70
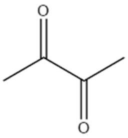	2,3-butanedione	2.06	2.13
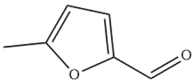	2-furancarboxaldehyde,5-methyl	5.61	1.41
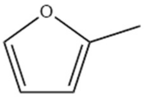	furan,2-methyl-	2.13	0.35
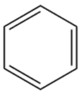	benzene	2.52	0.79
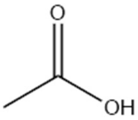	acetic acid	2.20	1.35
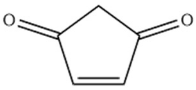	4-cyclopentene-1,3-dione	4.83	0.71
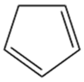	1,3-cyclopentadiene	1.89	0.37
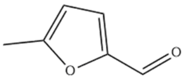	2-furancarboxaldehyde,5-methyl	5.11	0.99
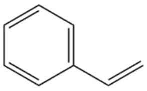	styrene	4.89	0.71
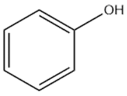	phenol	6.02	0.48

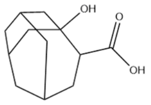	3-Hydroxy-tricyclo[4.3.1.1(3,8)] undecane-4carboxylic acid	3.65	0.58
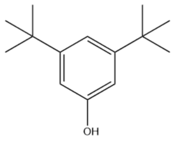	Phenol,3,5-bis(1,1-dimethylethyl)-	9.90	0.43
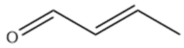	2-butenal, (E)-	2.44	0.41
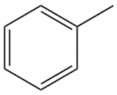	toluene	3.50	0.49
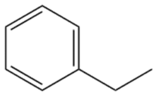	ethylbenzene	4.58	0.28
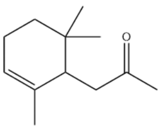	Cyclohexene, 1,5,5-trimethyl-6-acetylmethyl	7.06	0.36
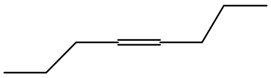	4-Octyne	11.25	2.70
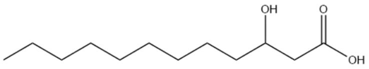	Dodecanoic acid, 3-hydroxy	8.04	0.55
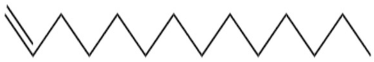	1-Tetradecene	7.60	0.10
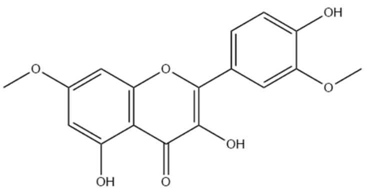	rhamnazin	7.15	0.28
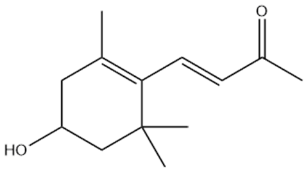	(3E)-4-(4-Hydroxy-2,6,6-trimethyl-1-cyclohexen-1-yl)-3-buten-2-one	6.50	0.61
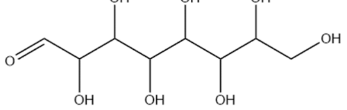	2,3,4,5,6,7,8-heptahydroxyoctanal	6.74	0.54

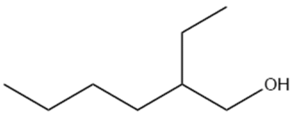	1-Hexanol,2-ethyl-	6.26	0.19
